# Direct current stimulation of endothelial monolayers induces a transient and reversible increase in transport due to the electroosmotic effect

**DOI:** 10.1038/s41598-018-27524-9

**Published:** 2018-06-18

**Authors:** Limary M. Cancel, Katherin Arias, Marom Bikson, John M. Tarbell

**Affiliations:** Department of Biomedical Engineering, The City College of New York, New York, USA

## Abstract

We investigated the effects of direct current stimulation (DCS) on fluid and solute transport across endothelial cell (EC) monolayers *in vitro*. Our motivation was transcranial direct current stimulation (tDCS) that has been investigated for treatment of neuropsychiatric disorders, to enhance neurorehabilitation, and to change cognition in healthy subjects. The mechanisms underlying this diversity of applications remain under investigation. To address the possible role of blood-brain barrier (BBB) changes during tDCS, we applied direct current to cultured EC monolayers in a specially designed chamber that generated spatially uniform direct current. DCS induced fluid and solute movement across EC layers that persisted only for the duration of the stimulation suggesting an electroosmosis mechanism. The direction of induced transport reversed with DCS polarity – a hallmark of the electroosmotic effect. The magnitude of DCS-induced flow was linearly correlated to the magnitude of the applied current. A mathematical model based on a two-pore description of the endothelial transport barrier and a Helmholtz model of the electrical double layer describes the experimental data accurately and predicts enhanced significance of this mechanism in less permeable monolayers. This study demonstrates that DCS transiently alters the transport function of the BBB suggesting a new adjunct mechanism of tDCS.

## Introduction

Transcranial direct current stimulation (tDCS) is being investigated for treatment of a broad range of neuropsychiatric disorders (e.g. depression, neuropathic pain, epilepsy^1^), as an adjunct to rehabilitation (e.g. stroke^2^, traumatic brain injury^3^), and to facilitate cognitive performance in healthy individuals (e.g. accelerated learning^4^). Validating the efficacy of tDCS and optimizing therapy dose, requires an understanding of the underlying mechanisms, including the cellular targets of tDCS – which cell types are directly activated. Efforts spanning decades^5^ have quantified the effects of DCS on neurons, even identifying which neurons^6^ and neuron compartments^7^ are more sensitive. A role for glia has also been proposed^8,9^. Here we address a novel cellular target for DCS - the endothelial cells (ECs) that form and regulate the blood-brain-barrier (BBB). We previously developed an *in vitro* cell culture model of BBB stimulation, where alternating current pulses mimicking deep brain stimulation protocols induced increases in water flux across EC monolayers through disruption of tight junctions between ECs^10^. In the present study, we adapt this system to examine the physiological effects of simulated tDCS protocols on endothelial transport properties.

There is a compelling body of indirect evidence suggesting BBB activation by tDCS. Imaging studies show tDCS changes cerebral perfusion in humans^11–13^ and animals^14^, though these data cannot distinguish between changes in BBB function secondary to neuronal stimulation or direct electrical stimulation of the BBB. The most evident physiological outcome of tDCS is skin vascular response reflected in erythema^5,15,16^, though current density is much higher at the skin. However, given a robust current-mediated skin vascular reaction during tDCS, it is reasonable to consider the possibility of such a change within the brain. Finally, long-duration/low-intensity electric fields have been shown to stimulate vascular endothelial growth factor production, and directed reorientation of ECs^17,18^. Therefore, the hypothesis of direct modulation of BBB function by direct current stimulation (DCS) is of interest.

A biophysical mechanism that could lead to transport of water and solutes across the BBB in response to an applied electric field is electroosmosis^19^. This process derives from the movement of charged ions through small channels or pores that have fixed charge on their surface. A layer of mobile charge (double layer) near the fixed charge is able to move in response to an electric field applied parallel to the surface. This mobile charge drags surrounding medium resulting in net fluid and associated solute transport through the pore. The direction of the net fluid flow can be reversed by changing the polarity of the applied electric field. Direct current produces a sustained direction of electroosmotic transport. The relative importance of electroosmosis is amplified in very small channels (micro, nano) where the hydrodynamic resistance limits pressure-driven convection. The paracellular transport pathways across endothelial layers typically have a fixed negative charge on their surface and are of nano-scale dimensions^20^ suggesting that electroosmosis may be an important mechanism of transport in the presence of an applied electric field. Sanchez *et al*. have provided evidence suggesting electroosmosis, arising as a result of spontaneously generated circulating currents, as a primary mechanism of fluid transport in the corneal epithelium^21^.

To begin to address the possible role of the BBB in tDCS efficacy and safety, we applied 10 minutes of DCS characteristic of tDCS to monolayers of cultured ECs and measured their transport barrier responses. We found that DCS produced significant stimulation-polarity-specific fluid and solute movement across the EC layer that persisted only as long as the current was applied and was enhanced in cells with tighter junctions – all characteristic of the electroosmotic effect. A mathematical model based on a two pore description of the endothelial transport barrier and a Helmholtz model of the electrical double layer describes the experimental data accurately and predicts enhanced significance of this mechanism in tighter (less permeable) monolayers.

## Materials and Methods

### Cell culture

Immortalized mouse brain endothelial cells, bEnd.3, were obtained from American Type Culture Collection (Manassas, VA) and cultured in Dulbecco’s modified Eagle’s medium (DMEM; Sigma, St. Louis, MO) supplemented with 10% fetal bovine serum (FBS; Hyclone, Logan, UT), 3 mM L-glutamine (Sigma, St. Louis, MO) and 1% penicillin-streptomycin (Sigma, St. Louis, MO). bEnd.3 s were plated at 6 × 10^4^ cells/cm^2^ in Transwell filters (1.1 or 4.67 cm^2^ membrane area, 0.4 μm pores; Corning, Lowell, MA) coated with fibronectin (Sigma, St. Louis, MO) and cultured for 4–5 days before transport experiments. Experimental media consisted of phenol-red free DMEM (Sigma, St. Louis, MO) supplemented with 1% bovine serum albumin.

### Measurement of solute and water flux

**Figure 1:**
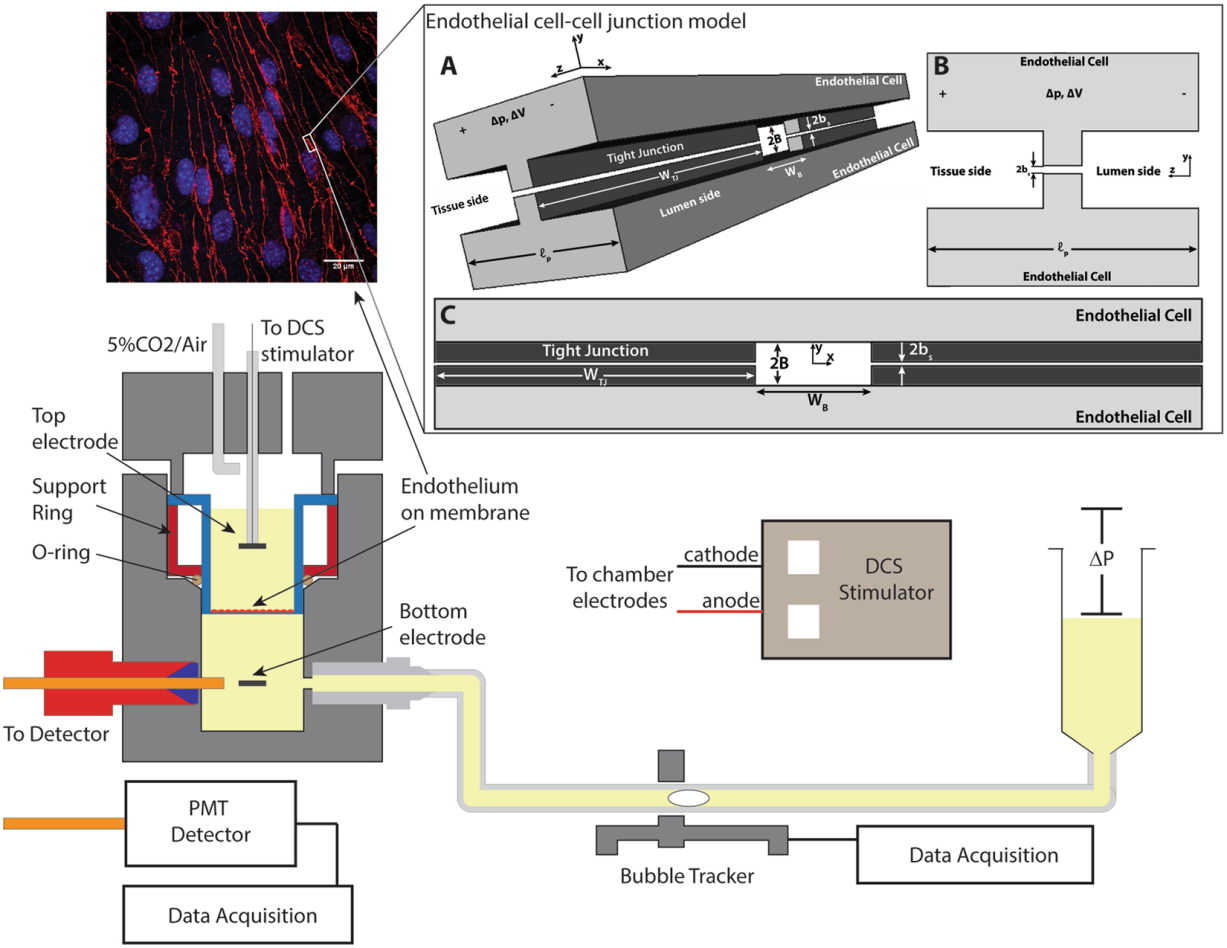
Schematic of the transport system used to apply direct current stimulation (DCS) to endothelial monolayers. Transwell filters containing the endothelial monolayer were sealed inside the chamber. The flux of a fluorescently tagged solute was measured through a fiberoptic cable coupled to a photon counting detector. Excitation light was provided by a laser connected to a second fiberoptic cable placed at 45° angle to the first (not shown). Water flux was measured via the automated bubble tracker, after the application of a 10 cm H_2_O hydrostatic pressure differential (ΔP). Endothelial monolayers were stimulated with 0.1–1.5 mA current for 10 minutes using a transcranial Direct Current Stimulator. Changes in water and solute flux across the monolayer were monitored during stimulation. TOP LEFT: bEnd.3 monolayer grown on Transwell filter and immunostained for the tight junction protein ZO-1. TOP RIGHT: Endothelial cell-cell junction model. (**A**) Three-dimensional sketch of the junction between two endothelial cells. The central orifice of height 2B and width W_B_ is the break in the tight junction (TJ) strand. The TJ has a narrow slit of height 2b_s_ and width W_TJ_ which allows current but not fluid flow. Volume flows in the z direction, from the lumen to the tissue side, as hydrostatic (ΔP) and electrical (ΔV) forces are applied. (**B**) Orthogonal view of yz plane. Note that the break in the TJ is not shown in this view. (**C**) Orthogonal view of yx plane.

Simultaneous measurement of solute and water flux was achieved with an apparatus developed in our lab (Fig. 1) and used in several previous studies of endothelial transport^22,23^. The apparatus was kept inside a Plexiglass hood and maintained at 37 °C. The flux of 70 kDa tetramethylrhodamine isothiocyanate-tagged dextran (Sigma, St. Louis, MO) or 5-carboxytetramethylrhodamine (TAMRA, 430 Da; Sigma, St. Louis, MO) was measured using an automated fluorometer system which has been described elsewhere^22^. Briefly, the Transwell filter containing a cell monolayer was sealed within a transport chamber, forming luminal and abluminal compartments. Transport of water and solutes occurred solely across the cell monolayer. The abluminal compartment was connected to a fluid reservoir via tygon tubing and borosilicate glass tubing. The reservoir could be lowered to apply a hydrostatic pressure differential across the monolayer. The concentration of fluorescently tagged solute in the abluminal compartment was measured with a Model D48 photon counting detector (C&L Instruments; Hershey, PA). Excitation light at 532 nm was provided by a 10-mW Crystal Laser. The FluorMeasure acquisition software and Model PC-DAQ control card (C&L Instruments) were used to control the laser exciter and detector, and for data acquisition.

Permeability was calculated as follows:

1 $$P=\frac{({\rm{\Delta }}{C}_{a}/{\rm{\Delta }}t)\times {V}_{a}}{{C}_{l}\times A}$$

where ΔC_a_/Δt is the change in abluminal concentration per unit time, V_a_ is the fluid volume in the abluminal compartment, C_l_ is the concentration in the luminal compartment (assumed to remain constant over the course of the experiment), and A is the area of the filter.

The water flux (J_v_) was measured by tracking the position of an air bubble that was inserted into the glass tubing using an automated bubble tracking system^24^. The bubble displacement was used to compute J_v_ values by

2 $${J}_{V}=\frac{{\rm{\Delta }}d}{{\rm{\Delta }}t}\times \frac{F}{A}$$

where Δd/Δt is the bubble displacement per unit time and F is a tube calibration factor equal to the fluid volume per unit length of tubing. Simultaneous solute and water flux experiments carried out in the apparatus described above (Fig. 1) used the small Transwell (1.1 cm^2^). To achieve lower current densities, a larger Transwell (4.67 cm^2^) was used in a similar apparatus equipped only to make water flux measurements using the same bubble tracker system described above.

### Direct current stimulation

Direct current stimulation was achieved by means of a pair of Ag/AgCl electrodes (4 mm × 1 mm disk; A-M Systems, Sequim, WA) positioned 6 mm above and below the cell monolayer. On experiments using the large Transwell membrane, Ag/AgCl electrodes were 8 mm × 1 mm disks positioned 14 mm above and below the cell monolayer. A transcranial Direct Current Stimulator (model 1300-A, Soterix Medical, New York) was used to apply 0.1–1.5 mA current across the monolayers for 10 minutes. The current was ramped up to the final value over 30 seconds and ramped down to zero over 30 seconds. This current level and duration are typical of tDCS applications in patients with neurological disorders^1^.

In a typical experiment, the Transwell filter was rinsed twice with experimental medium before being inserted into the transport chamber. A solution of 5 μg/mL of 70 kDa dextran or 0.65 μM TAMRA was added to the luminal compartment. Each transport experiment began by lowering the abluminal reservoir to apply a 10 cm H_2_O pressure differential (to drive convective flux). Data were collected for 60 min to obtain baseline values of the water and solute fluxes. For convective experiments, DCS (1 mA) was then immediately applied for 10 min, and water and solute flux data were collected under the combined influence of the pressure differential and DCS. After DCS application, data were collected for up to 120 min under convective conditions. For diffusive experiments, after 60 min of convection the abluminal reservoir was raised to its original level and the flux was allowed to equilibrate for 30 min before application of DCS for 10 min. After DCS application, data were collected for up to 120 min under diffusive conditions.

### Immunostaining of bEnd.3 monolayer with ZO-1

Following some experiments, bEnd.3 monolayers were immunostained for the tight junction protein ZO-1 to assess monolayer integrity. After 60 min of convective flux, control (no DCS) and DC-stimulated monolayers were washed with PBS and fixed in 1% paraformaldehyde (PFA; Polysciences Inc., Warrington, PA) for 10 min. Monolayers were then permeabilized with 0.2% Triton X-100 for 10 min, blocked with 10% goat serum and 0.1% Triton X-100 for 1 hr, and incubated with primary rabbit anti-ZO-1 antibody (1:200; Invitrogen, Rockford, IL) overnight at 4 °C. After washing 5X with PBS, ZO-1 was detected with AF555 goat anti-rabbit IgG secondary antibody (1:500; Life Technologies Inc, Eugene, OR) for 60 min at room temperature. After washing 4X with PBS, monolayers were counterstained with DAPI nuclear stain and mounted with Fluoromount (Southern Biotech, Birmingham, AL). Imaging was conducted in a Zeiss LSM 510 Confocal Laser Scanning system at 40X/oil magnification.

### Mathematical model of electroosmosis

To interpret the experimental transport data, a mathematical model of electroosmosis was developed.

#### Model geometry

We use the simplified structure for the junction between two endothelial cells proposed in Fu *et al*.^25^. The junction strand is idealized as periodic units consisting of a large pore and a continuous narrow slit. A single unit is shown in Fig. 1 (top right insert). The tight junction (TJ) has a narrow slit of height 2b_s_ and width W_TJ_ which allows current but not fluid flow. The central orifice of height 2B and width W_B_ is the break in the TJ strand and allows both fluid and current flow.

#### Model description

Charge separation that occurs naturally at biological interfaces leads to the formation of an electrical double layer. Electroosmotic flow is caused by the force induced by an electrical field on the mobile portion of the double layer. The derivation of the flow and current through the junction follows that presented by Grodzinsky^19^ for a cylindrical pore, but with the 2-pore slit geometry shown in Fig. 1. We assume that the two types of pores (TJ and break) are arranged in parallel and there is no interaction between them, i.e., there is no current or fluid flow in the lateral x direction (Fig. 1C). If the liquid in the pores is treated as a Newtonian fluid, and inertial forces are neglected, the governing equation for fluid flow in the presence of an electrical field is

3 $$-\nabla p+\mu {\nabla }^{2}{\bf{v}}+{\rho }_{e}{\bf{E}}=0$$

where p is the pressure, v is the velocity, ρ_e_ is the charge density, and **E** is the applied electrical field. In the z-direction, we assume fully developed flow such that v = v_z_(y), ρ_e_ = ρ_e_(y) and ${\rm{\partial }}p/{\rm{\partial }}z={\rm{\Delta }}p/{\ell }_{p}$, where Δp is the pressure drop across the pore and ${\ell }_{p}$ is the length of the pore (thickness of EC in the junction region), as shown in Fig. 1. We also assume that the y-component of the electrical field, which is due to the electrical double layer, is much greater than the applied electrical field and, therefore, unaffected by it (see Appendix). Then, the governing equation is written as

4 $$\frac{{\rm{\Delta }}p}{{\ell }_{p}}=\mu \frac{{{\rm{\partial }}}^{2}{v}_{z}}{{\rm{\partial }}{y}^{2}}+\varepsilon \frac{{\rm{\Delta }}V}{{\ell }_{p}}\frac{{{\rm{\partial }}}^{2}{\rm{\Phi }}(y)}{{\rm{\partial }}{y}^{2}}$$

where ε is the permittivity of the fluid, ΔV is the potential drop across the length of pore, and Φ(y) is the electrical double layer potential as a function of distance from the wall. Equation (4) can be integrated twice using the boundary conditions ${\rm{\partial }}{v}_{z}/{\rm{\partial }}y={\rm{\partial }}{\rm{\Phi }}/{\rm{\partial }}y\equiv 0$ at y = 0 by symmetry, and v_z_ = 0, ${\rm{\Phi }}\equiv \zeta$ at the mechanical slip plane y =  ±(B − δ) to obtain an expression for the velocity

5 $${v}_{z}=\frac{({y}^{2}-{(B-\delta )}^{2})}{2\mu {\ell }_{p}}{\rm{\Delta }}p+\frac{\varepsilon (\zeta -{\rm{\Phi }}(y))}{\mu {\ell }_{p}}{\rm{\Delta }}V$$

ζ is the potential at the slip plane, the so-called zeta potential, which occurs not at the wall but several water molecule diameters away from the wall at y = B − δ. After substituting for the potential function, Φ(y) (see Appendix), Equation (5) can be integrated over the cross-sectional area of a large break pore to obtain the volume flow through the pore

6 $${Q}_{B}=-\frac{2{B}^{3}{W}_{B}}{3\mu {\ell }_{p}}{\rm{\Delta }}p+\frac{2\varepsilon \zeta B{W}_{B}}{\mu {\ell }_{p}}{\rm{\Delta }}V$$

where W_B_ is the width of the break in the TJ strand. To relate this equation to the flux data obtained, we first define the total number of pores as

7 $${n}_{B}=\frac{{A}_{m}}{{W}_{B}}{L}_{jt}(1-\,f)$$

8 $${n}_{TJ}=\frac{{A}_{m}}{{W}_{TJ}}{L}_{jt}\,f$$

where n_B_ is the number of large break pores in the junction strand, n_TJ_ is the number of narrow slit pores in the strand, L_jt_ is the total length of the junction strand per unit area, A_m_ is the area of the monolayer, and $f$ is the fraction of L_jt_ that is tight junction (narrow slit). Multiplying equations (6) by (7) and rearranging we obtain an equation for J_v_:

9 $${J}_{v}=-\frac{2{B}^{3}{L}_{jt}(1-f)}{3\mu {\ell }_{p}}{\rm{\Delta }}p+\frac{2\varepsilon \zeta B{L}_{jt}(1-f)}{\mu {\ell }_{p}}{\rm{\Delta }}V$$

To calculate ΔV from the known applied current density (J_i_) we first obtain an expression for the current through each pore, which can be defined in terms of a sum of a pressure-driven flow term and a conduction term^19^ (see Appendix):

10 $$\begin{array}{c}{i}_{B}=\frac{2\varepsilon \zeta B{W}_{B}}{\mu {\ell }_{p}}{\rm{\Delta }}p-[\frac{2B{W}_{B}\sigma }{{\ell }_{p}}+\frac{4{W}_{B}{\varepsilon }^{2}{\zeta }^{2}}{\mu {\ell }_{p}d}]{\rm{\Delta }}V\\ {i}_{TJ}=-\frac{2{b}_{s}{W}_{TJ}\sigma }{{\ell }_{p}}{\rm{\Delta }}V\end{array}$$

where i_B_ is the current through the break pore, i_TJ_ is the current through the TJ pore, σ is the fluid conductivity, and d is the Debye length (see Appendix). For our slit geometry, assuming the pores form a parallel circuit, the total applied current (i_TOTAL_) is equal to the sum of the currents across all the pores. Using equations (7) and (8), and noting that J_i_ = i_TOTAL_/A_m_, we obtain the following expression for the total applied current density:

11 $${J}_{i}=\frac{2\varepsilon \zeta B{L}_{jt}(1-f)}{\mu {\ell }_{p}}{\rm{\Delta }}p-[\frac{2B\sigma }{{\ell }_{p}}+\frac{4{\varepsilon }^{2}{\zeta }^{2}}{\mu {\ell }_{p}d}]\,{L}_{jt}(1-f)\,{\rm{\Delta }}V-\frac{2{b}_{s}\sigma {L}_{jt}\,f}{{\ell }_{p}}{\rm{\Delta }}V$$

The anatomical cell parameters and fluid properties in equations (9) and (11) are either known, can be calculated or be estimated. Table 1 shows the values used in our calculation. Finally we define the electroosmostic flow enhancement, E, as follows

12 $$E=1+\frac{{J}_{v}^{DCS}}{{J}_{v}^{{\rm{\Delta }}p}}=1+\frac{-3\varepsilon \zeta }{{B}^{2}}\frac{{\rm{\Delta }}V}{{\rm{\Delta }}p}$$

where J_v_^DCS^ is the flow induced by the application of DCS (the second term on the right hand side of equation (9)) and J_v_Δ^p^ is the flow induced by the pressure gradient (first term on the right hand side of equation (9)). To fit our data to the model, the baseline J_v_ data is first used to estimate B in equation (9) when ΔV = 0. Then equations (11) and (9) are used to calculate E, with ζ as the parameter varied to optimize the coefficient of determination (R^2^) for the model.

**Table 1:** Parameters used in the model.

Anatomical Parameters	Value	Source
Thickness of EC, ${\ell }_{p}$	700 nm	^66^
Height of break in TJ, 2B	25 nm	calculated from J_v_ for each data point, average value shown
Height of TJ, 2b_s_	2 nm	^25,67^
Width of break in TJ, W_B_	150 nm	^67^
Width of TJ, W_TJ_	2490 nm	^67^
Junction length per unit EC monolayer area, L_jt_	1300 cm/cm^2^	measured in present study
Fraction of junction length that is TJ, $f$	0.94	$f=\frac{{W}_{TJ}}{{W}_{TJ}+{W}_{B}}$
Area of EC monolayer, A_m_	1.1 cm^2^	Transwell growth area
**Fluid Properties**		
Viscosity, μ	0.00078 Pa/s	^68^
Permittivity, ε	7.08 × 10^−10^ F/m	^69^
Conductivity, σ	1.5 S/m	^69^
**Applied Forces**		
Pressure drop, Δp	980.7 N/m^2^	applied in present study
Current density, J_i_	9.1 A/m^2^	applied in present study

### Statistical analysis

Data are presented as mean and standard deviation (SD). Statistical analysis was performed using either paired or unpaired t-tests with p < 0.05 considered significant. Where multiple comparisons were made (e.g. for comparisons of baseline, DCS and post-DCS data) Bonferroni’s correction was used to reduce the p-value considered significant to 0.5 divided by the number of comparisons (e.g. for three comparisons p < 0.016 was considered significant). GraphPad Prism was used to perform statistical analysis and to generate data plots.

### Data availability

All data generated or analysed during this study are included in this published article (and its Supplementary Information files).

## Results

### Effect of DCS polarity and current on water flux

**Figure 2:**
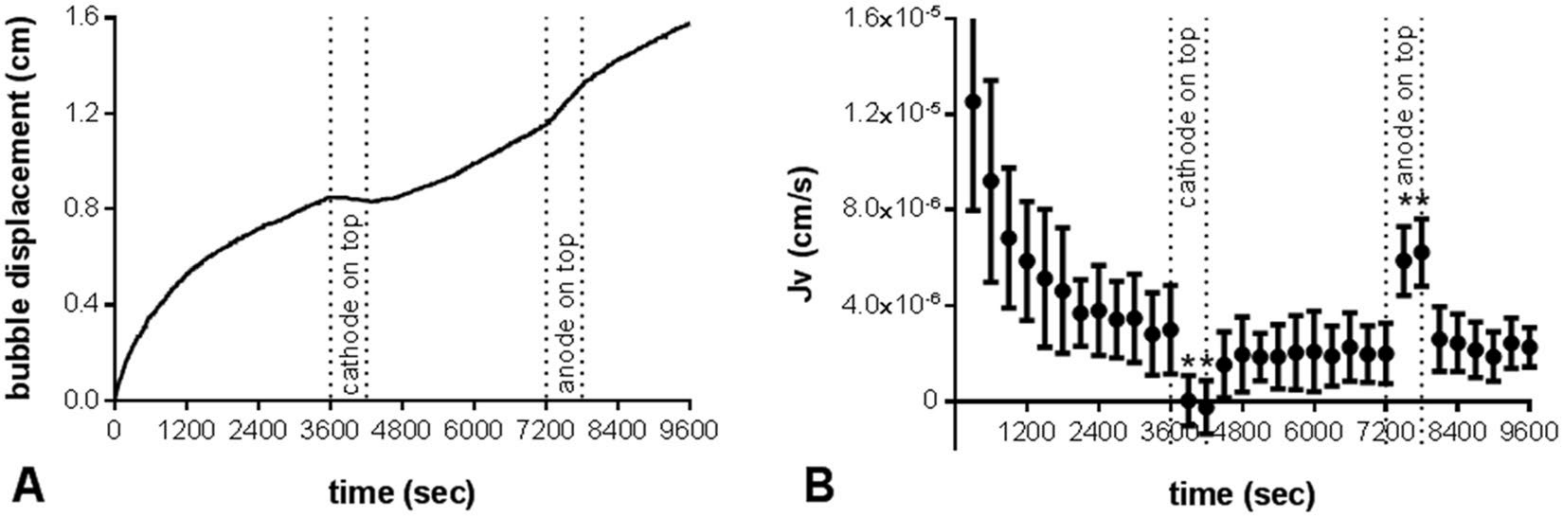
Effect of reversing DCS polarity on water flux. (**A**) Bubble displacement data from single experiment. bEnd.3 monolayers were first exposed to a 10 cm H_2_O hydrostatic pressure gradient to establish the baseline J_v_. DCS (1 mA) was then applied at t = 3600 sec with the cathode on top inducing an immediate decrease in flux that persisted for the 10 min duration of DCS. After reestablishing the baseline for 3600 sec, DCS was applied with the anode on top (t = 7200 sec) inducing an immediate increase in flux that persisted for the duration of DCS. (**B**) Average J_v_ as a function of time calculated from the raw data at intervals of 300 sec for n = 5 experiments performed as in (**A**). J_v_ significantly decreased or increased with DCS application in the B-A or A-B mode, respectively. *p = 0.007 for B-A mode and p = 0.0002 for A-B mode vs baseline 5 minutes before DCS application by paired t-test.

J_v_ was measured before, during, and after application of 10 min of 1 mA DCS from the basal to apical side of the monolayer (B-A polarity, cathode on top of monolayer) and apical to basal (A-B polarity; anode on to top of monolayer) directions (Fig. 2A). Application of DCS produced an immediate and transient response in J_v_. The direction of change in J_v_ was dependent on the polarity of the applied electric field, while its magnitude was independent of the polarity (Fig. 2B). For the bEnd.3 monolayers in Fig. 2B, an average decrease in J_v_ of 3.10 × 10^−6^ cm/s (SD 1.31 × 10^−6^) in the B-A direction was not significantly different from an average increase in J_v_ of 4.05 × 10^−6^ cm/s (SD 0.76 × 10^−6^) in the A-B direction. Both changes were significantly different from the baseline J_v_ calculated 5 minutes before DCS application (P = 0.007 and P = 0.0002, respectively).

**Figure 3:**
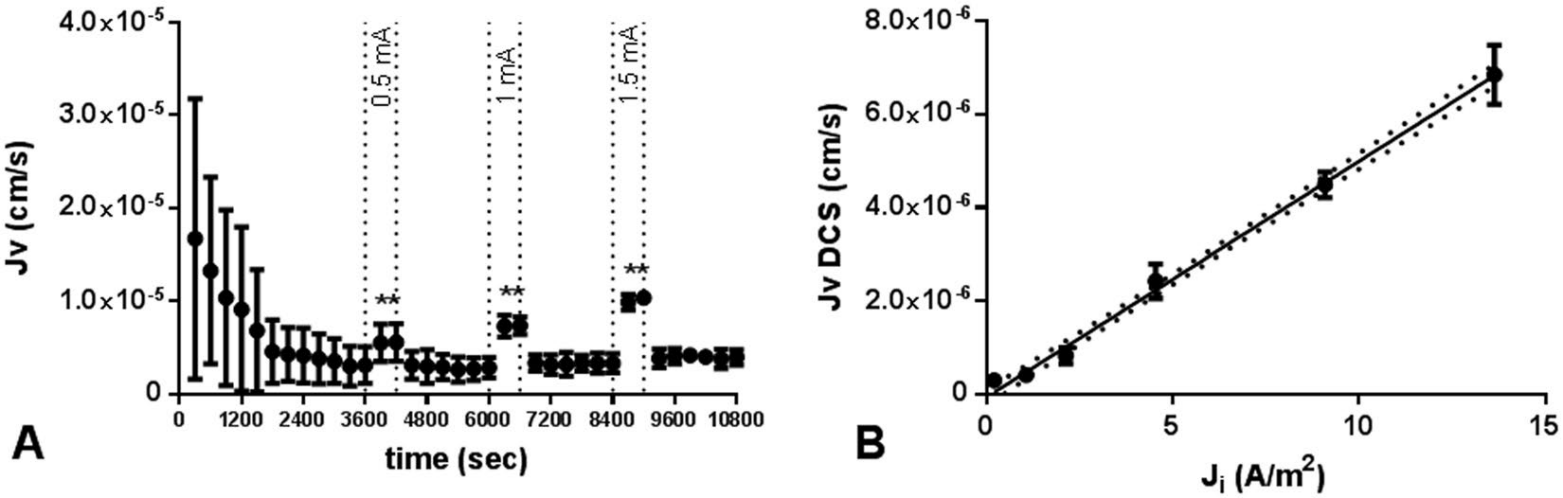
Effect of DCS current magnitude on water flux. (**A**) Average J_v_ as a function of time for n = 6 experiments performed as follows: bEnd.3 monolayers were first exposed to a 10 cm H_2_O hydrostatic pressure gradient to establish the baseline J_v_. DCS was then applied at t = 3600 sec at a current of 0.5 mA for 10 min. After reestablishing the baseline for 30 min, DCS was applied at a current of 1 mA for 10 min. Finally, the baseline was reestablished for 30 min and DCS was applied at a current of 1.5 mA for 10 min. (**B**) DCS-induced flow (J_v_^DCS^) was found to be linearly correlated with the magnitude of the applied current density (J_i_). Dotted lines show 95% confidence interval; R^2^ = 0.98. *p = 1.8 × 10^−5^ for 0.5 mA; p = 1.9 × 10^−7^ for 1 mA; p = 1.4 × 10^−6^ for 1.5 mA vs baseline 5 minutes before DCS application by paired t-test.

Application of DCS at increasing current magnitude (0.5, 1, and 1.5 mA; current density (J_i_) = 4.54, 9.09, and 13.64 A/m^2^, respectively) induced changes in J_v_ of increasing magnitudes (2.43 × 10^−6^cm/s (SD 0.44 × 10^−6^), 4.49 × 10^−6^ cm/s (SD 0.39 × 10^−6^), and 6.85 × 10^−6^ cm/s (SD 0.71 × 10^−6^), respectively; Fig. 3A). After each DCS application, J_v_ returned to the baseline value. To evaluate whether the effect extended to lower current densities, current magnitudes of 0.1, 0.5, and 1 mA where applied to a larger Transwell (4.67 cm^2^) corresponding to J_i_ of 0.21, 1.07, and 2.14 A/m^2^ respectively. These lower current densities resulted in statistically significant increases in J_v_ (2.61 × 10^−7^ cm/s (SD 1.56 × 10^−7^; P = 0.009), 3.58 × 10^−7^ cm/s (SD 1.7 × 10^−7^; P = 0.001), and 8.31 × 10^−7^ (SD 1.75 × 10^−7^; P = 2 × 10^−7^), respectively; Fig. 3B). The magnitude of the DCS-induced flow (J_v_^DCS^) showed a strong linear correlation with the magnitude of the applied current density (R^2^ = 0.98; Fig. 3B).

**Figure 4:**
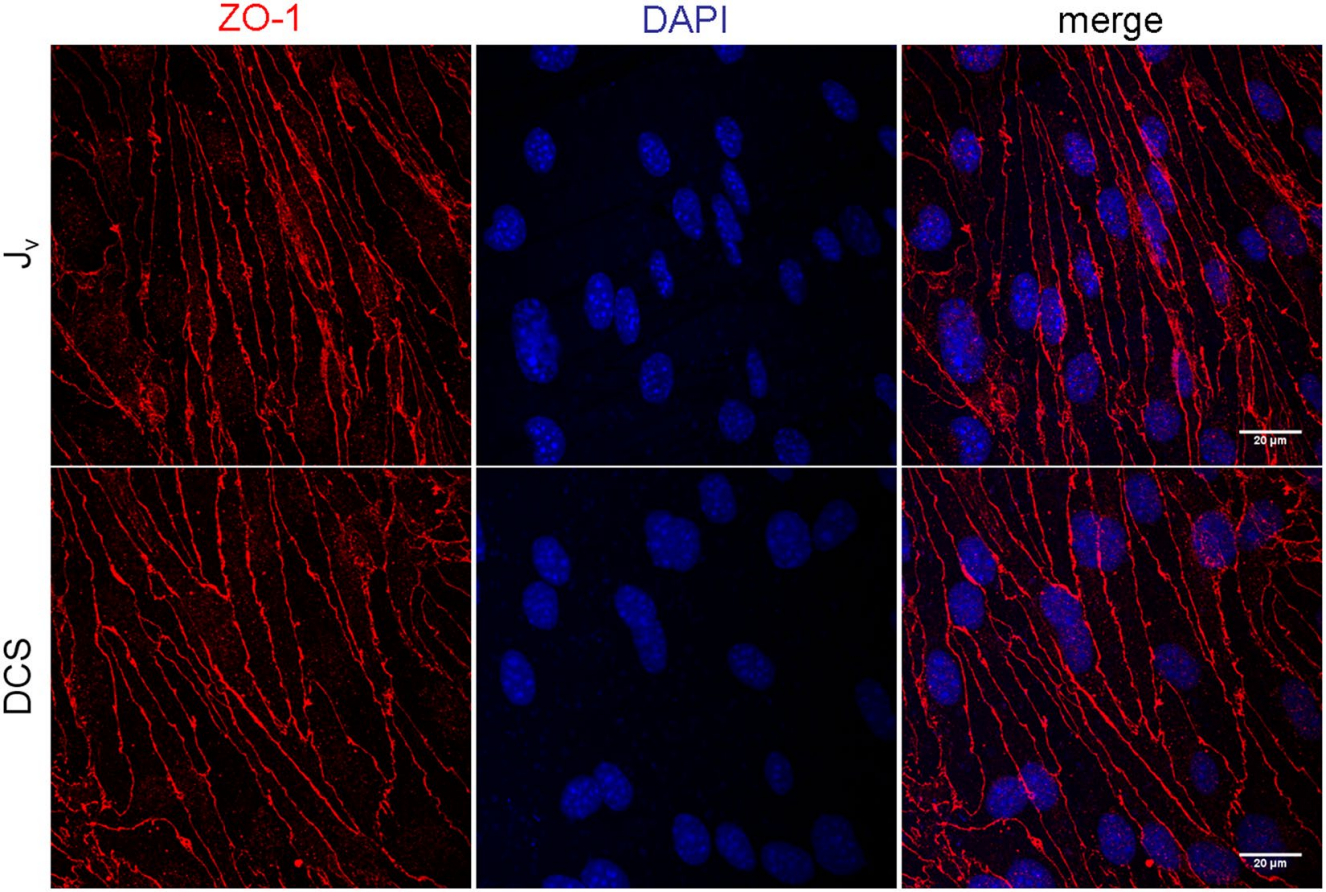
Immunostaining of bEnd.3 monolayer with ZO-1 antibody. bEnd.3 monolayers were exposed to 1 hr of 10 cm H2O hydrostatic pressure gradient (J_v_), or 1 hr of 10 cm H_2_O hydrostatic pressure gradient +10 min of DCS (DCS). Afterwards the monolayers were immunostained for the TJ protein ZO-1 (red). The nucleus was counterstained with DAPI (blue). No damage to the monolayer integrity was observed after DCS. These images were also used to estimate the length of the junction per unit area (L_jt_ in Table 1).

The rest of the experiments in this study were carried out at 1 mA in the 1.1 cm^2^ Transwell and with the A-B direction to drive the flow in the same direction as the ΔP-induced flow, i.e. from apical to basal side of monolayer, which is the physiological direction in which ECs experience flow. Visual inspection and immunostaining of the monolayers showed no damage to the monolayer integrity after DCS (Fig. 4), a fact supported by the immediate recovery of baseline J_v_ after DCS is terminated.

### Effect of DCS on water and solute flux

**Figure 5:**
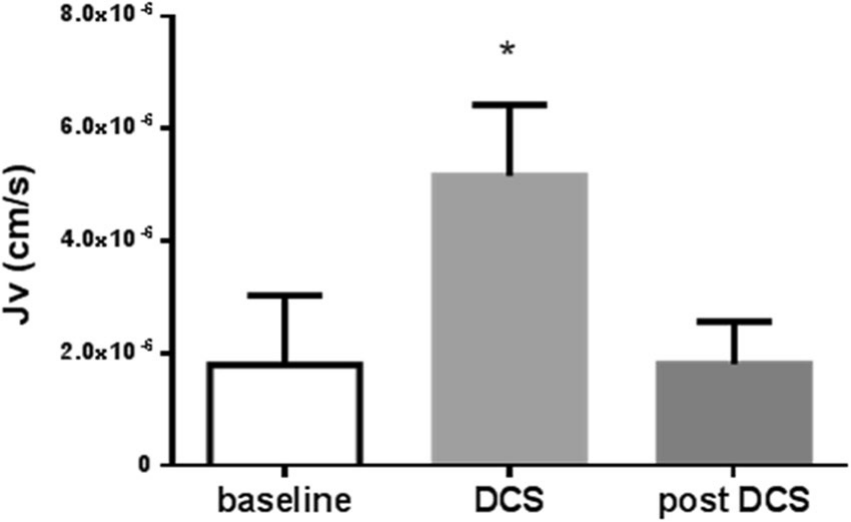
Effect of 1 mA DCS on J_v_ across bEnd3 monolayers. N = 7; p = 3.1 × 10^−5^ vs baseline by paired t-test.

The effect of 1 mA DCS on J_v_ across bEnd.3 monolayers is summarized in Fig. 5. bEnd.3 monolayers had baseline J_v_ of 1.79 × 10^−6^ cm/s (SD 1.24 × 10^−6^), consistent with previous measurements^26^. Upon application of 1 mA DCS, bEnd.3 monolayers experienced an immediate significant increase in water flux (2.88-fold, p = 3.1 × 10^−5^). J_v_ after DCS is turned off was not significantly different from baseline (p = 0.97).

**Figure 6:**
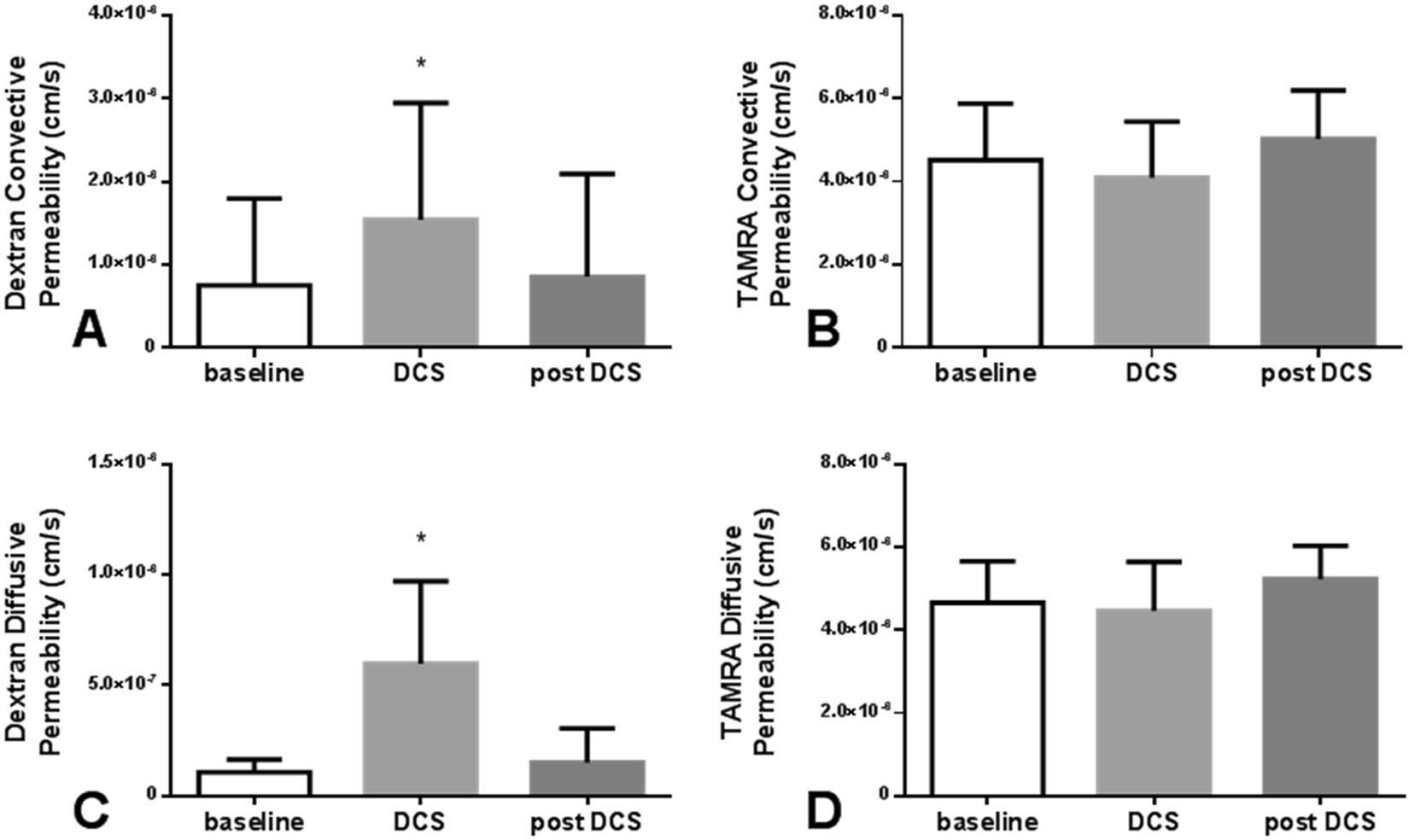
Effect of 1 mA DCS on bEnd3 permeability to Dextran 70 kDa (**A**,**C**) and TAMRA (**B,D**) under convective (**A**,**B**) or diffusive (**C**,**D**) conditions. (**A**) Dextran permeability increased by 2-fold under convective conditions and (**C**) by 5.5-fold under diffusive conditions (**C**). There was no increase in TAMRA permeability under either condition (**B**,**D**). N = 12 for (**A**); N = 13 for (**B**); N = 8 for (**C**) and (**D**). *p = 7 × 10^−5^ in (**A**) and p = 0.004 in (**B**) vs baseline by paired t-test.

Figure 6 summarizes the effects of DCS on bEnd.3 permeability to 70kDa-Dextran and TAMRA. Under convective conditions, i.e. with a hydrostatic pressure gradient driving flow that can convect solute, bEnd.3 monolayers had baseline permeabilities of 7.5 × 10^−7^ cm/s (SD 1.0 × 10^−6^) for 70kDa-Dextran and 4.5 × 10^−6^ cm/s (SD 1.4 × 10^−6^) for TAMRA (Fig. 6A,B). DCS application increased bEnd.3 permeability to 70kDa-Dextran (by 2-fold to 1.5 × 10^−6^ cm/s (SD 1.4 × 10^−6^); p = 7 × 10^−5^), but had no effect on the permeability of TAMRA (p = 0.41). Under diffusive conditions with no pressure driven flow, DCS increased 70kDa-Dextran permeability by 5.5-fold (p = 0.004) from 1.1 × 10^−7^ cm/s (SD 5.7 × 10^−8^) to 6.0 × 10^−7^ cm/s (SD 3.7 × 10^−7^) but had no effect (p = 0.47) on TAMRA permeability (Fig. 6C,D).

### Model Results

**Figure 7:**
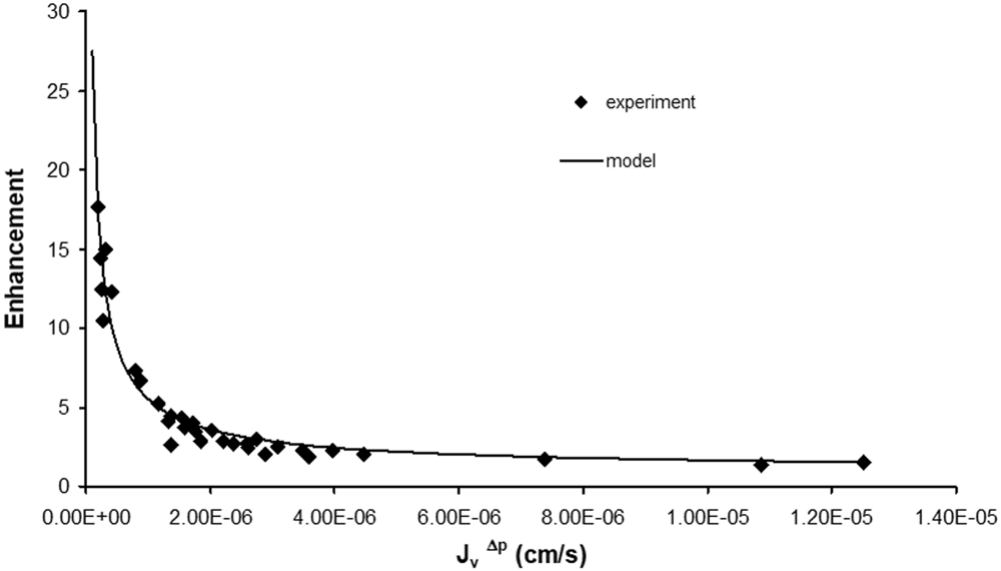
Electroosmotic Enhancement. Equations 11 and 12 were used to model the eletroosmotic enhancement for bEnd.3 monolayers. The model (solid line) is in good agreement with the experimental values (◆) with R^2^ = 0.94.

Figure 7 shows electroosmotic enhancement predictions of the model along with the experimental values obtained at the same current density. The model is in excellent agreement with the experimental data, with an R^2^ of 0.94. The value of ζ used to optimize the fit, −20.5 mV, is in the range of values previously measured for biological materials such as mouse neuroblastoma cells, −15 mV^27^, and rat organotypic hippocampal cultures, −22 mV^28^. The data and the model show the strong trend of greater enhancement for tighter monolayers (lower J_v_^Δp^).

## Discussion

The goal of this study was to isolate and quantify the effects of DCS on endothelial layers that model the BBB. To this end, we exposed bEnd.3 monolayers grown on Transwell membranes to a low level of direct current of (1 mA) for 10 minutes (typical of tDCS human trials^29^) and measured water and solute flux before, during, and after DCS. We report that DCS induced an immediate increase in water flux that persisted during DCS, and immediately returned to baseline value when DCS was turned off. We also demonstrated that the direction of water flow could be reversed by reversing the direction of the applied current (Fig. 2). In addition, we showed that the magnitude of DCS-induced flow is linearly correlated to the applied current density, and that the effect persists at current densities experienced in the brain during tDCS^30,31^ (Fig. 3).

These results point to an electroosmotic mechanism of action. Electroosmotic flow is the motion of fluid induced by an applied electrical potential across a fluid channel that contains an electrical double layer^19^. Electroosmotic flow is most significant in smaller channels where pressure driven flow is low due to higher hydrodynamic resistance (Equation 12; Fig. 7).

Solute flux experiments using 70 kDa Dextan and TAMRA (430 Da) showed significant increases in the permeability of the larger solute, while there was no measureable increase in the permeability of the smaller solute. The relative increase in permeability (for 70 kDa Dextran) was even greater when the experiment was carried out under diffusive conditions (no applied pressure differential). We can infer from these results that the effect of DCS on solute permeability is due to an increase in convection through the large pores (breaks in the TJ) rather than an increase in the number or width of the pores. If there had been an increase in the total number of pores, we would expect to see an increase in the permeability of the small solute, whose transport is diffusion dominated.

A mathematical model of electroosmotic flow was developed using an idealized two pore geometry for the junction between two endothelial cells and a Helmholtz model for the electrical double layer. The model accurately replicated the experimental data (Fig. 7). In our analysis, we have used an idealized model of the nanoscale features of the cell-cell junction to calculate the potential drop, ΔV, across the EC monolayer (Equation 11). Using the macroscale electrical properties of the EC monolayer, ΔV could also be defined as

13 $${\rm{\Delta }}V={i}_{TOTAL}\times {R}_{TOTAL}={J}_{i}\times TEER$$

where i_TOTAL_ is total applied current, and R_TOTAL_ is the total resistance of the EC monolayer. TEER is the transendothelial electrical resistance, defined as R_TOTAL_ multiplied by the area of the monolayer (A_m_), and is a parameter widely used to characterize the barrier properties of endothelial monolayers^32^. Substituting this equation for ΔV in Equation (12), it is apparent that monolayers with higher TEER, i.e. tighter monolayers, would experience higher flow enhancement at the same applied current density and pressure drop:

14 $$E=1+\frac{-3\varepsilon \zeta }{{B}^{2}}\frac{{J}_{i}}{{\rm{\Delta }}p}TEER$$

The use of a cultured endothelial cell monolayer does not fully reflect the physiological behavior of the intact BBB. However, a recent *in vitro* study of co-culture models of the BBB that included astrocytes and basement membrane components along with ECs (bEnd.3) demonstrated that co-culture components did not significantly reduce the hydraulic conductivity or solute permeability compared to EC monoculture, and found cultured bEnd.3 to be a good *in vitro* model for transport of large solutes when compared to *in vivo* models^26^. An additional limitation of our study is that solute flux experiments were conducted at a current density (9.09 A/m^2^) larger than what brain tissue is predicted to experience (0.2 A/m^2^)^30,31^. However, since the solute flux data shows that the effect on solute flux is due to the DCS-induced increase in convective flow, and we have shown that DCS-induced flow persists at current densities similar to those experienced in the brain (Fig. 3B), it is reasonable to expect that solute effects would be present at current densities lower than used in the present study, and that these effects would scale with current density magnitude.

It should also be noted that computational models used to predict current density in the brain due to tDCS do not include the vasculature and its potential contribution to current distribution and local current density. These models predict electric field and current density for the ‘bulk’ brain extracellular space. If one assumes blood vessels are transparent to current flow, then the current density across the BBB would be the same as bulk brain. More likely, as the resistivity of the BBB and blood is different from brain, microscopic current flow will be altered. We hypothesize that as blood is more conductive than brain parenchyma, the vasculature provides a relatively attractive path for current flow. Depending on the vascular morphology (capillary organization), current will cross in and out of the BBB at specific points, and at these locations current density may be several fold higher than in the bulk brain, corresponding to the higher current densities tested here. Interestingly, even for the same current density as bulk, the electric field across the BBB during tDCS would be orders of magnitude higher than bulk because of the resistivity of the BBB. Our experimental system applies stimulation directly across the BBB model–very different than tDCS (*in vivo*) or brain slice models. For these reasons current density (not electric field) is reasonable to control and report, and values tested here span a range of potential *in vivo* tDCS values. For similar reasons, the use here of “anode on top” or “cathode on top” to describe directionality across the Transwell (BBB model) should not be confused with anodal or cathodal tDCS describing direction across the bulk brain^33^. The polarity dependence of tDCS is complex^7,34–36^ and additional analysis would be needed before speculating on the role of asymmetric vascular morphology^37^ in mediating any such dependence^30^.

We also note that microvessels in the BBB are expected to provide a much tighter transport barrier than our cultured monolayers. We have measured the TEER of cultured bEnd.3 monolayers and found values of ~40 Ωcm^2^. Measurements in rat cerebral microvessels *in vivo* show an average TEER of order 1000 Ωcm^2,^^38^. Based on the relationship between TEER and electroosmotic flow enhancement (Equation 14) we can expect large increases in water and solute flux in BBB microvessels *in vivo*, even at a reduced current density. According to our theory, change in transport during DCS electroosmosis is linear with stimulation intensity (Equation 14; Fig. 3) and there is no threshold for inaction.

Although we have shown that the effects of DCS on endothelial layers can occur independent of direct neuron stimulation, changes in BBB function may influence neuronal activity and plasticity. The main effect found in the present study is an increase in water flux upon DCS application. At the lowest current density tested (0.214 A/m^2^), the induced flux was ~2.6 × 10^−7^ cm/s (Fig. 3B), similar to estimates of interstitial flux in brain parenchyma (~3 × 10^−7^ cm/s^39^). Increased interstitial flow around neurons, driven by DCS-induced increase in water flux across the BBB may affect neurons directly through the fluid shear stress imposed upon them or indirectly by the clearance of local metabolites. Recent studies of the enhancement of brain function by elevated interstitial flow during sleep support this idea^40^. Studies in other cell types, such as smooth muscle cells and fibroblasts^41^, and cancer cells^42^, have shown that very low levels of interstitial flow can affect gene and protein expression as well as physiological function through fluid shear stress. The degenerin channels of C. elegans touch receptor neurons displayed a rapid response to imposed laminar shear stress^43^. Shear stress increased intracellular Ca^2+^ levels in cultured adult astrocytes^44^.

In addition, the ECs themselves are exposed to increased interstitial shear stress as a result of the increased water flux through their junctions. It is well established that ECs modulate their gene and protein expression in response to fluid shear stress on their apical surface^45,46^. Most notably, shear stress upregulates the endothelial nitric oxide synthase (eNOS) gene^47^, protein expression^48^, and the production of the neurotransmitter nitric oxide (NO) by ECs^49,50^. But increased flow through EC junctions also increases NO production^51^.

In conclusion, this is the first study to isolate and explain a significant response of ECs to low levels of DCS. We have shown that exposing endothelial monolayers to DCS induces an increase in water and solute flux. This increase is immediate, transient, and reversible, consistent with electroosmosis as the mechanism of action. These findings reveal a new mechanism that may contribute to the aggregate changes in neuronal activity produced by tDCS. Given the central role of transport across the BBB in brain function, the significant and reversible changes in transport reported here, could influence cellular and network function through a cascade of mechanisms^52–57^. Many of the metabolic, chemical, and neurophysiological consequences of tDCS^58–65^, and thus behavioral/clinical outcomes, could theoretically be explained by BBB stimulation. The cellular targets of tDCS remain to be investigated; this paper shows EC stimulation is a new plausible target.

## Appendix

This appendix describes details of the mathematical formulation of the electroosmosis model and assumptions used to solve it.

### Electrical Force Density

The electrical force density in the z-direction, ((ρ_e_**E**)_z_) in Equation 3, can be obtained using Gauss’s law to substitute for ρ_e_

A1 $${({\rho }_{e}{\bf{E}})}_{z}=(\nabla \cdot \varepsilon {\bf{E}}){E}_{z}=(\frac{{\rm{\partial }}(\varepsilon {E}_{y})}{{\rm{\partial }}y}){E}_{z}$$

where ε is the permittivity of the fluid. We then assume that E_y_ is solely due to the ionic atmosphere and is unaffected by fluid flow in the pore

A2 $${E}_{y}=\frac{{\rm{\partial }}{\rm{\Phi }}(y)}{{\rm{\partial }}y}$$

where Φ(y) is the electrical double layer potential. The applied electrical field, E_z_, can be written in terms of the potential drop, ΔV, across the length of the pore, ${\ell }_{p}$, such that Equation A1 becomes

A3 $${({\rho }_{e}{\bf{E}})}_{z}=\varepsilon \frac{{\rm{\Delta }}V}{{\ell }_{p}}\frac{{{\rm{\partial }}}^{2}{\rm{\Phi }}(y)}{{\rm{\partial }}{y}^{2}}$$

Substituting Equation A3 into the governing equation (Equation 3) yields, in the z-direction,

A4 $$\frac{{\rm{\Delta }}p}{{\ell }_{p}}=\mu \frac{{{\rm{\partial }}}^{2}{v}_{z}}{{\rm{\partial }}{y}^{2}}+\varepsilon \frac{{\rm{\Delta }}V}{{\ell }_{p}}\frac{{{\rm{\partial }}}^{2}{\rm{\Phi }}(y)}{{\rm{\partial }}{y}^{2}}$$

The boundary conditions are:

A5 $$\begin{array}{rcl}{\frac{{\rm{\partial }}{v}_{z}}{{\rm{\partial }}y}|}_{y=0} & = & 0\\ {\frac{{\rm{\partial }}{\rm{\Phi }}}{\partial y}|}_{y=0} & = & 0\\ {v}_{z}(B{\rm{-}}\delta ) & = & 0\\ {\rm{\Phi }}(B-\delta ) & = & 0\end{array}$$

Integrating A4 twice and applying A5 yields an equation for the velocity in the z-direction

A6 $${v}_{z}=\frac{({y}^{2}-{(B-\delta )}^{2})}{2\mu {\ell }_{p}}{\rm{\Delta }}p+\frac{\varepsilon (\zeta -{\rm{\Phi }}(y))}{\mu {\ell }_{p}}{\rm{\Delta }}V$$

### Helmholtz double layer

As in^19^, we assume a Helmholtz double layer in order to obtain analytical solutions for the volume flow through a break pore, Q_B_, and the current flow through break and TJ pores, i_B_ and i_TJ_, respectively. This leads to an approximation of the potential function as^19^

A7 $${\rm{\Phi }}(y)\cong \{\begin{array}{c}0\\ \frac{\zeta [y-(B-d)]}{d}\end{array}\,\,\begin{array}{c}0\le y\le B-d\\ (B-d)\le y\le B\end{array}$$

where d is the thickness of the double layer (equal to the Debye length (d = 1/κ)), and it’s been assumed that d << B. Note that the y-origin is placed in the middle of the slit (Fig. 1C), and the function Φ(y) has been written for the positive half of the slit. The Debye length is given by

A8 $$\frac{1}{\kappa }=\sqrt{\frac{\varepsilon RT}{2{z}^{2}{F}^{2}{c}_{i0}}}\equiv {\bf{Debye}}\,{\bf{length}}$$

where R is the universal gas constant, T is the temperature, z is the ion valence, F is the Faraday constant, and c_i0_ is the bulk concentration of the ith species. Equation A8 assumes a single symmetrical electrolyte. For the case of the culture media used in the present study (DMEM), the major electrolyte is NaCl (c_i0_ = 0.1197 M). At T = 310 K, using Equation A8, d = 0.9 nm.

### Volume flow

The volume flow through a break pore can be obtained by first substituting Equation A7 for the double layer potential into the velocity equation (Equation 5) and then integrating over the cross-sectional area to obtain the flow though half the slit pore. By symmetry, the flow though the entire pore becomes

A9 $${Q}_{B}=2{W}_{B}[{\int }_{0}^{B}\frac{{\rm{\Delta }}p}{2\mu {\ell }_{p}}({y}^{2}-{(B-\delta )}^{2})dy+{\int }_{0}^{B}\frac{\varepsilon \zeta {\rm{\Delta }}V}{\mu {\ell }_{p}}dy-{\int }_{B-d}^{B}\frac{\varepsilon {\rm{\Delta }}V}{\mu {\ell }_{p}}\frac{\zeta [y-(B-d)]}{d}dy]$$

where the third integral recognizes that Φ(y) = 0 in the range 0 ≤ y ≤ B-d. Using the assumption that B >> d >> δ, Equation A9 reduces to Equation 6.

### Current flow

The current flow through each type of pore is defined in terms of sum of conduction and flow terms^19^

A10 $${i}_{B}=2{W}_{B}{\int }_{0}^{B}[{\rho }_{e}(y){v}_{z}(y)-\sigma (y)\frac{{\rm{\Delta }}V}{{\ell }_{p}}]dy$$

A11 $${i}_{TJ}=2{W}_{TJ}{\int }_{0}^{B}[-\sigma (y)\frac{{\rm{\Delta }}V}{{\ell }_{p}}]dy$$

For the small TJ pore (narrow slit in Fig. 1), we assume no flow occurs due to high hydrodynamic resistance (v_z_ = 0) so that only the conduction term remains. Although the fluid conductivity (σ(y)) can generally vary with y, we assume it to be uniform over the pore cross section. Using Equations A1-A2 to substitute for ρ_e_ in terms of Φ(y), then subsequently using Equation A7 to substitute for Φ(y), and assuming that B >> d >> δ, Equations A10 and A11 reduce to Equation 10.
